# Supplementary material for: Homology-mediated transformation of frog-killing fungus Batrachochytrium dendrobatidis illuminates chytrid development and pathogenesis
Source: Proc Natl Acad Sci U S A. 2025 Oct 28;122(44):e2507572122. doi: 10.1073/pnas.2507572122 (PMC12595416; doi:10.1073/pnas.2507572122)
Supplement: Supplementary file 1 — Appendix 01 (PDF) [file pnas.2507572122.sapp.pdf]

PNAS

Supporting Information for

Homology-mediated transformation of frog-killing fungus Batrachochytrium dendrobatidis illuminates chytrid development and pathogenesis

Stephanie M. Brody[1#], Erik Kalinka[1#], Sarah Prostak[1], Tamilie Carvalho[2,3], Jarrett Man[1], Timothy Y. James[2], and Lillian K. Fritz-Laylin[4*]

[1] Department of Biology, University of Massachusetts, Amherst, MA, 01003, United States

[2] Department of Ecology and Evolutionary Biology, University of Michigan, Ann Arbor, MI 48109, United States

[3] Institute for Global Change Biology, School for Environment and Sustainability, University of Michigan, Ann Arbor, MI, 48109, USA

[4] Howard Hughes Medical Institute and the Department of Biology, University of Massachusetts, Amherst, MA, 01003, United States

[#] These authors made equal contributions to the manuscript

[*]Email correspondence to: lfritzlaylin@umass.edu

This PDF file includes:

Figures S1 to S3
Tables S1 to S3
Legends for Movies S1
Legends for Datasets S1 to S3

Other supporting materials for this manuscript include the following:

Movies S1
Datasets S1 to S3

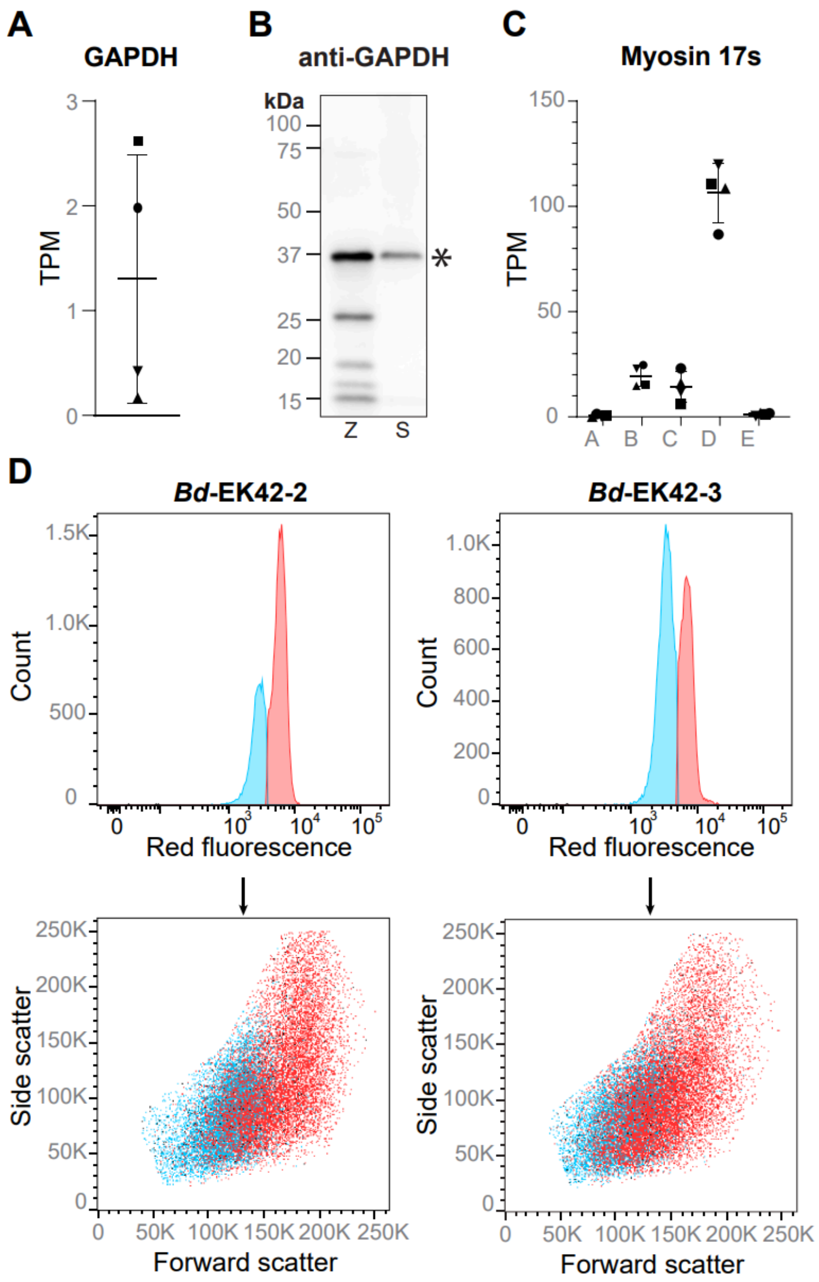

Fig. S1. Additional information about *Bd* gene expression and fluorescence of transformed cell lines. (A) Transcripts per million (TPM) of GAPDH in WT JEL423 zoospores. **(B)** Immunoblot analysis of GAPDH expression in WT JEL423 zoospores (Z) and 24 hour sporangia (S). Star denotes expected size band size for native GAPDH protein (36 kDa). **(C)** TPM of the 5 Myosin 17s in WT JEL423 zoospores. In the current *Bd* JEL423 genome, Myo17B is split across two gene annotations, separating the motor domain from the chitin synthase domain. Here, Myo17B TPM was based on the myosin motor domain annotation alone. **(D)** Backgating of putative subpopulations GAPDH-mRuby3 cells onto forward scatter vs. side scatter shows the two distinct fluorescence peaks emerged from differently sized subpopulations. Scatter plots show single cells only, after gating for cells and doublet discrimination: mRuby3 negative cells shown in black, weakly fluorescent in blue, strongly fluorescent in red..

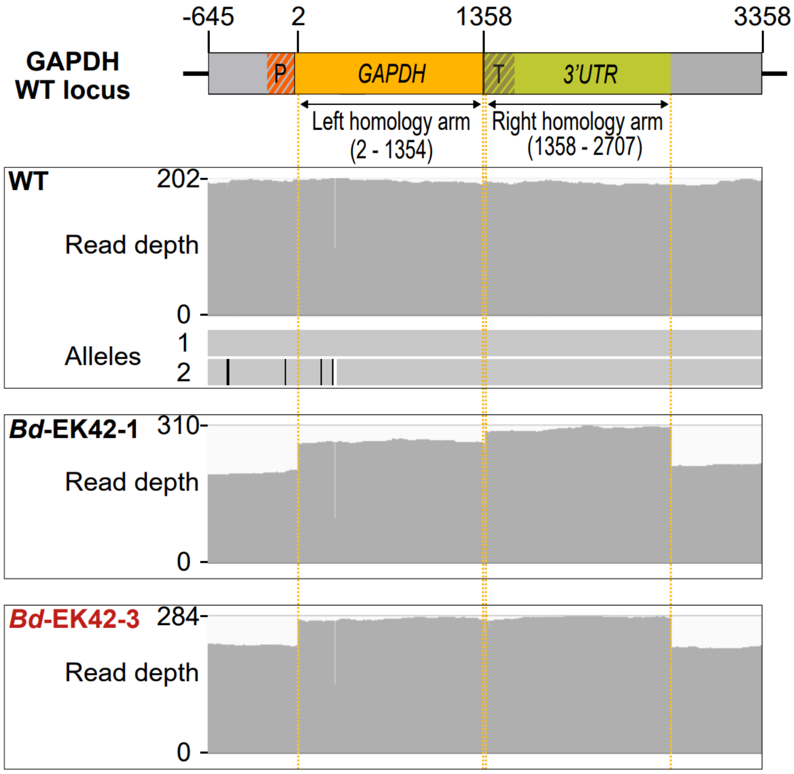

Fig. S2. GAPDH wild type (WT) locus structure and read depths according to whole genome assemblies. Haplotype resolution was achieved through SNPs (black lines) and a 3 bp deletion (white line) specific to allele 2. Read depth shows the number of reads that map to wild type (WT) and edited loci. The transgenic clones show higher read depth in the region with sequence identity to the homology arms, supporting the presence of additional copies of the homology arm sequences in these strains.

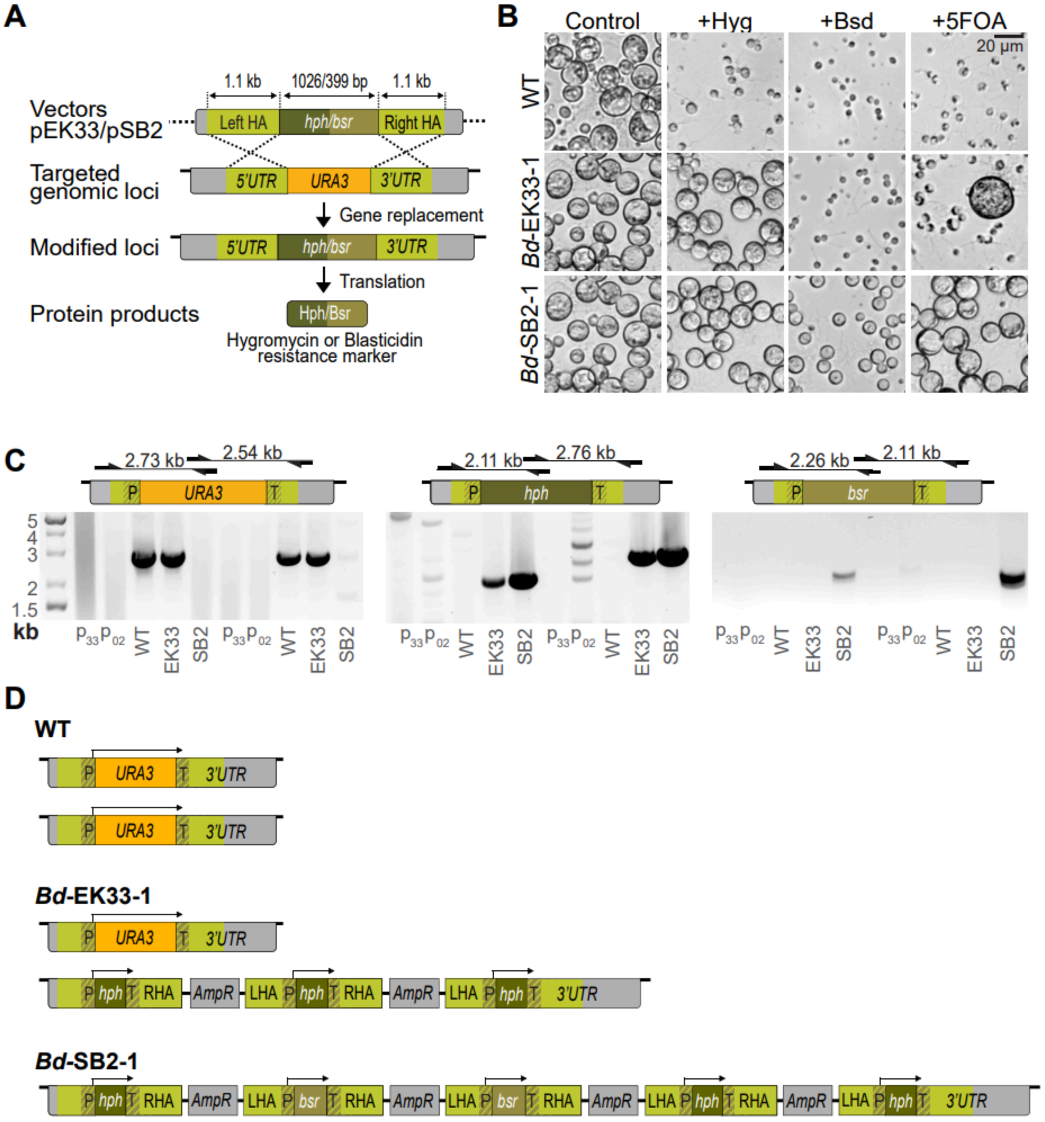

Fig. S3. Gene replacement of *Bd URA3* by sequential transformation with two selection constructs: (A) Schematic representation of the experimental strategy used to generate stable *BdURA*3 knock-out through sequential gene replacement using homologous recombination. Cells were sequentially transformed with the vector pEK33 then pSB02 (*hph:* hygromycin resistance gene, bsr: blasticidin resistance gene, HA: homology arm). **(B)** Bright field images of wild type (WT), *Bd*-EK33-1, and *Bd*-SB02-1 lines cells grown in media supplemented with hygromycin B, blasticidin S or 5-FOA for 3 days. **(C)** (Top) Diagrams showing primer locations and amplicon sizes of different primer pairs to native *URA3* locus or transformed loci. (Bottom) Gel electrophoresis of genotyping PCR confirming presence of the native *URA3* locus and *hph* or *bsr* locus specific integration into WT, *Bd*-EK33-1 (EK33), and *Bd*-SB02-1(SB2) lines (P_{33} = pEK33 plasmid control, P_{02}=pSB02 plasmid control). **(D)** Diagram of reconstructed alleles of the *URA3* loci of WT, *Bd*-EK33-1 and *Bd*-SB02-1 based on whole genome sequencing assembly results. Note: WGS of *Bd*-SB02-1 recovered only a single allele of the *URA3* locus, but read depth analysis indicates that there are likely two copies of this allele present in the genome. The arrows above each allele denote predicted open reading frames (P: promoter, T: terminator, *hph*: hygromycin resistance gene, *bsr*: blasticidin resistance gene, L/RHA: left/right homology arm).

Table S1: Overview of electroporation-based stable *Bd* transformation. A general timeline outlining the various steps involved in generating transgenic *Bd* lines using gene targeting vectors.

Day	Procedure	Details
1	Electroporate cells	
1-5	Recover electroporated cells in non-selective liquid media	
2	Inoculate antibiotics selection test	Inoculate 1 out of 7 wells for each sample
5	Assess antibiotics containing well for transient growth	Check for growing sporangia and release of motile zoospores
5	Plate samples onto selection and control agar plates	One of each type of plate per sample
9-21	Check selection plates for transient transformants and control plates for cell survival	
23-26	Inspect selection plates for stable growth (>100 swimming zoospores per field of view)	Required incubation duration varies between transformation trials and gene targeting vectors
26-50	Single colony isolation	Two consecutive rounds
50-55	Culture amplification	
From 92	Cryopreservation and downstream analysis	

Table S2. Statistics on transformation success. Statistics for the generation of stable fluorescent transformants of *Bd* JEL423 using gene targeting vectors.

Transformed plasmid	Transformation #	Days until formation of lawn on selection plate (x = did not grow)	Fluorescence	Strains isolated from transformation
pEK33	1	19	N/A	
	2	18	N/A	*Bd*-EK33-1
	3 (linearized plasmid)	19	N/A	*+/-ura3*
	4 (linearized plasmid)	12	N/A	*-/-ura3*
pEK42	1	x	--	
	2	x	--	
	3	19	Yes	*Bd*-EK42-1, *Bd*-EK42-2, *Bd*-EK42-3
pEK48	1	30	No	
	2	33	No	
	3	19	Yes	*Bd*-EK48-1, *Bd*-EK48-2
pSB02	1 (linearized plasmid)	18	N/A	*Bd*-SB02-1
	2 (linearized plasmid)	14	N/A	

Table S3. ***Bd*** **strains available from the Collection of Zoosporic Eufungi at the University of Michigan (CZEUM).** Accessible at czeum.herb.lsa.umich.edu.

Strain name	Parent strain	Transformed plasmid	Target locus (NCBI locus tag)	Expressed recombinant protein(s)
JEL423-FLL	-	-	-	-
Bd-EK33-1	JEL423-FLL	pEK33	URA3, replacement (BDEG_24941)	hph-G4S-cMyc (HygR)
Bd-EK42-1	JEL423-FLL	pEK42	GAPDH, 3' end (BDEG_26645)	hph (HygR)
Bd-EK42-3	JEL423-FLL	pEK42	GAPDH, 3' end (BDEG_26645)	*BdGAPDH-G4S-mRuby3, hph (HygR)*
Bd-EK48-2	JEL423-FLL	pEK48	Myo17D, 3' end (BDEG_23276)	*BdMyo17D-G4S-mRuby3, hph (HygR)*
Bd-SB02-1	*Bd-EK33-1*	pSB02	URA3, replacement (BDEG_24941)	hph-G4S-cMyc (HygR), bsr (BsdR)
+/-ura3	JEL423-FLL	pEK33	URA3, replacement (BDEG_24941)	hph-G4S-cMyc (HygR)
-/-ura3	JEL423-FLL	pEK33	URA3, replacement (BDEG_24941)	hph-G4S-cMyc (HygR)

Movie S1 (separate file). Video of Myo17D-mRuby3 localization during mucin-induced encystation shown in Fig. 4G. Images were taken every 5 seconds in DIC and mRuby3. Time is displayed in minutes:seconds.

Dataset S1 (separate file). ***Batrachochytrium dendrobatidis*** **gene targeting vectors used in this study.** This data set lists the names, source, and cloning strategy used for each plasmid. The JEL423 genome assembly (GCA_000149865.1) was used to construct gene models.

Dataset S2 (separate file). Primers used in PCR screenings. Dataset containing primers used in this study.

Dataset S3 (separate file). Assemblies of relevant loci. Sequences of assembled loci relevant to this study.
